# Thiamine disulfide derivatives in thiol redox regulation: Role of thioredoxin and glutathione systems

**DOI:** 10.1002/biof.2121

**Published:** 2024-09-20

**Authors:** Alessandra Folda, Valeria Scalcon, Federica Tonolo, Maria Pia Rigobello, Alberto Bindoli

**Affiliations:** ^1^ Department of Biomedical Sciences University of Padova Padova Italy; ^2^ Department of Comparative Biomedicine and Food Science University of Padova Legnaro Italy; ^3^ Institute of Neuroscience (CNR) University of Padova Padova Italy

**Keywords:** fursultiamine, glutathione and thioredoxin systems, sulbutiamine, thiamine, thiamine disulfide, thiol redox control

## Abstract

Thiamine (vitamin B1), under the proper conditions, is able to reversibly open the thiazole ring, forming a thiol‐bearing molecule that can be further oxidized to the corresponding disulfide. To improve the bioavailability of the vitamin, several derivatives of thiamine in the thioester or disulfide form were developed and extensively studied over time, as apparent from the literature. We have examined three thiamine‐derived disulfides: thiamine disulfide, sulbutiamine, and fursultiamine with reference to their intervention in modulating the thiol redox state. First, we observed that both glutathione and thioredoxin (Trx) systems were able to reduce the three disulfides. In particular, thioredoxin reductase (TrxR) reduced these disulfides either directly or in the presence of Trx. In Caco‐2 cells, the thiamine disulfide derivatives did not modify the total thiol content, which, however, was significantly decreased by the concomitant inhibition of TrxR. When oxidative stress was induced by *tert*‐butyl hydroperoxide, the thiamine disulfides exerted a protective effect, indicating that the thiol form deriving from the reduction of the disulfides might be the active species. Further, the thiamine disulfides examined were shown to increase the nuclear levels of the transcription factor nuclear factor erythroid 2 related factor 2 and to stimulate both expression and activity of NAD(P)H quinone dehydrogenase 1 and TrxR. However, other enzymes of the glutathione and Trx systems were scarcely affected. As the thiol redox balance plays a critical role in oxidative stress and inflammation, the information presented can be of interest for further research, considering the potential favorable effect exerted in the cell by many sulfur compounds, including the thiamine‐derived disulfides.

## Graphical abstract

Modulation of thiol redox signaling by thiamine disulfide derivatives depends on the relative efficiency of glutathione and thioredoxin systems.

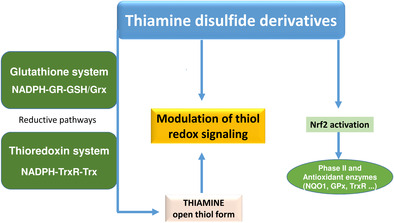

## 1 INTRODUCTION

Thiamine (vitamin B1) is the precursor of the coenzyme diphosphothiamine, playing a critical role in energy metabolism. The biochemical properties of diphosphothiamine, acting in decarboxylation and transketolase reactions, are well‐known.^1^ However, it has been acknowledged for a long time that thiamine, sometimes at relatively high doses largely beyond the established nutritional request, can be useful in diseases possibly not directly linked to its coenzymatic function.^2^, ^3^, ^4^, ^5^ It was also observed that vitamin B1 deficiency causes alterations to the nervous system and heart, linked to oxidative stress.^6^, ^7^, ^8^, ^9^ For instance, excessive alcohol consumption, such as chronic alcoholism, is responsible for brain damage and a decrease of thiamine levels in a process dependent, at least in part, on oxidative stress.^10^ More recently, Pavlova et al.^11^ evaluated the relationship between thiamine deficiency and oxidative stress in the brain and blood of thiamine‐deficient rats. The authors found that, in addition to an increased expression of thiamine transporter 1 (THTR1) and decreased expression of thiamine phosphokinase (TPK1), the thiamine deficiency‐dependent oxidative stress triggered an increase in reactive oxygen species (ROS) production and oxidation of free ‐SH groups, associated to an irreversible accumulation of diphosphothiamine in the disulfide form. The latter finding points out the strict correlation existing between oxidative stress and thiamine disulfide species formation. On the other hand, oxidative stress, and in general oxidants, appear involved in the direct alteration of thiamine, particularly in slightly alkaline conditions. Several oxidants such as hypoiodite, hypochlorite, hydrogen peroxide, ferricyanide,^12^ radiation^11^, ^13^ and, notably, reactive oxygen and nitrogen species^14^, ^15^, ^16^ were found to react with thiamine, leading to several products, some of which have potentially new bioactive properties. An interesting feature of thiamine resides in the thiazole moiety that, in slight alkaline conditions, can undergo a reversible ring opening leading to a thiol form.^12^, ^17^ Therefore, thiamine might be considered an incipient thiol,^18^ which is not only able to revert to its closed form, but also is endowed with all the properties of a ‐SH group‐bearing molecule. Although pH is strictly controlled in biological systems, it can be subjected to small changes in specific subcellular sites, such as in functioning mitochondria where the matrix pH is around 8.^19^ Once formed, the thiamine‐derived thiol can undergo oxidation to both unmixed and mixed disulfides, as previously observed.^20^, ^21^ This property, associated with the reversibility of the thiazole ring opening and closing process, was exploited to improve the bioavailability of thiamine as its intestinal absorption by the high affinity carriers exhibits a low turnover.^12^, ^22^ Therefore, several derivatives in the form of thioesters or disulfides were prepared, and they resulted in being taken up by the intestine more efficiently than thiamine.^4^, ^12^, ^21^ In fact, they are more lipophilic than thiamine and are not subjected to the action of thiaminase.^23^ Compared to thiamine, thiamine disulfide exhibits more favorable characteristics regarding antiflogistic and analgesic action and growth stimulation in bacteria.^12^ Recently, it has been reported^24^ that, in streptozotocin‐treated rats, thiamine disulfide (TSS) significantly reduces hyperglycemia and increases insulin sensitivity. This antidiabetic effect is associated with an increased expression of the genes involved in pancreatic insulin secretion.^24^ Isobutyryl thiamine disulfide (sulbutiamine, SBT) has been shown to hold antioxidant and nootropic effects in addition to improving asthenic syndrome.^25^, ^26^ Also, this molecule has been proposed for the treatment of cancer and some infections^25^ and it was shown to protect from hyperglycemia‐induced testicular damage.^27^ Potential beneficial therapeutic actions of thiamine tetrahydrofurfuryl disulfide (fursultiamine, FST) have been reported,^28^ and it was observed that this compound protected mouse cochlear hair cells from the damage induced by cisplatin and kanamycin by exerting antioxidants and antiapoptotic effects.^29^

On the basis of the above reported considerations, it appeared of interest to examine the pathways of reduction of the oxidized disulfide form and of other disulfide derivatives of thiamine and their potential effects on the cellular signaling pathways. In the present work, we have studied the behavior of thiamine disulfide, sulbutiamine, and fursultiamine, first examining their capability to undergo enzymatic reduction by the thioredoxin (Trx) and glutathione/glutaredoxin (Grx) systems and then their influence on cell functions, with particular reference to the Kelch‐like ECH‐Associating protein 1 (Keap1)‐nuclear factor erythroid 2 related factor 2 (Nrf2)‐antioxidant response element (ARE) pathway. We found that, in addition to being reduced by both the glutathione and Trx systems, they may also interact with the Nrf2 signaling pathway, bringing to the expression of genes involved in the detoxification and antioxidant processes.

## 2 EXPERIMENTAL PROCEDURES

### 2.1 Estimation of thioredoxin and glutathione reductase activities

Highly purified cytosolic TrxR (TrxR1) was prepared from rat liver according to Luthman and Holmgren.^30^ The protein content of the purified enzyme preparations was measured according to Lowry et al.^31^ Glutathione reductase (GR) from baker's yeast was purchased from Sigma‐Aldrich (St. Louis, MO, USA); Grx and Trx were from IMCO Corporation (Stockholm, Sweden). TrxR activity was determined by estimating the nicotinamide adenine dinucleotide phosphate, reduced form (NADPH) consumption at 340 nm for 30 min at 37°C, using a Lambda 2 spectrophotometer (PerkinElmer, Waltham, MA, USA). Aliquots of highly purified TrxR (72 nM) were tested in 0.2 M sodium phosphate buffer (pH 7.4) and 5 mM EDTA in the presence of 0.25 mM NADPH and 1 mM thiamine disulfide derivatives. When indicated, 7 μM Trx was added. GR activity was measured in the same buffer used for TrxR estimation and followed spectrophotometrically at 340 nm. Aliquots of 70 nM GR were incubated at 37°C in the presence of 0.25 mM NADPH and 0.1 mM reduced glutathione (GSH). The assay was started by the addition of 1 mM thiamine disulfide derivatives. When indicated, 40 nM Grx was added.

### 2.2 Cell cultures

For cellular studies, Caco‐2 cells (human colon‐rectal adenocarcinoma) were cultured at 37°C and 5% CO_2_ atmosphere in Dulbecco's Modified Eagle's Medium (DMEM) supplemented with 10% fetal bovine serum (FBS), 10,000 units/mL of penicillin, and 1 mg/mL of streptomycin.

### 2.3 Viability assay

Cell viability was determined with the 3‐[4,5‐dimethylthiazol‐2‐yl]‐ 2,5‐diphenyltetrazolium bromide (MTT) reduction assay.^32^ Cells (1 × 10^4^ cells/well) were seeded in a 96 well plate and treated with increasing concentrations of the thiamine disulfide derivatives. After 24 h, cells were washed with Phosphate‐Buffered Saline (PBS) and then incubated with 0.5 mg/mL MTT (Sigma‐Aldrich, St. Louis, MO, USA) for 3 h at 37°C. Afterward, 100 μL of stop solution (90% isopropanol/10% dimethyl sulfoxide) were added, and 15 min later, the absorbance (Abs) at 595 and 690 nm was estimated using a Tecan Infinite® M200 PRO plate reader (Tecan, Mannedorf, CH).

### 2.4 Enzyme activities in cell lysates

Caco‐2 cells were seeded (4 × 10^5^ in a six well plate) and treated with 100 μM thiamine disulfide derivatives for 24 h. Then, cells were harvested and washed with PBS twice. Each sample was lysed with a modified radioimmunoprecipitation assay (RIPA) buffer composed of 150 mM NaCl, 50 mM Tris/HCl, 1 mM ethylene diamine tetraacetic acid (EDTA), 1% Triton X‐100, 0.1% SDS, 0.5% sodium deoxycholate, 1 mM NaF, and 0.1 mM phenylmethylsulfonyl fluoride (PMSF), containing an antiprotease cocktail (Complete, Roche, Mannheim, DE). After 40 min at 4°C, the lysates were centrifuged at 15,800g to discard the debris, and the supernatant was tested for enzymatic activities. Total TrxR and GR activities were measured according to Tonolo et al.^33^ For TrxR activity, 50 μg of proteins from cell lysates were tested at 25°C in 0.2 M sodium phosphate buffer (pH 7.4) containing 5 mM EDTA and 20 mM 5,5′‐dithiobis(2‐nitrobenzoic acid) (DTNB). After 2 min, 0.25 mM NADPH was added, and the reaction was followed spectrophotometrically at 412 nm for about 10 min. GR activity of cell lysates (80 μg of proteins) was measured in 0.2 M Tris/HCl buffer (pH 8.1) containing 1 mM EDTA and 0.25 mM NADPH. The assay was started with 1 mM glutathione disulfide (GSSG) and monitored spectrophotometrically at 340 nm and at 25°C.

Grx activity was measured as described by Mieyal et al.^34^ The reaction mixture contained 0.2 mM NADPH, 0.5 mM GSH, 0.2 M Na‐K‐Pi buffer (pH 7.4), 0.4 units of GR, 5 mM EDTA, and 50 μg of cell lysates in a final volume of 1 mL. Reaction was carried out at 25°C with 2 mM hydroxyethyl disulfide (HEDS). The decrease of NADPH absorbance at 340 nm was monitored. Glutathione peroxidase (Gpx) activity was estimated as a decrease of NADPH absorbance at 340 nm using 200 μg of cell lysates in 50 mM Hepes/Tris (pH 7.0), 5 mM EDTA, 0.25 mM NADPH, 4 mM GSH, and 0.20 mM *tert*‐butyl hydroperoxide (TbOOH) at 25°C, according to Little et al.^35^

### 2.5 Western blot analysis

Cell lysates, obtained as described above, were utilized for monitoring the expression level of the various enzymes by Western blot (WB) analysis. Samples (30 μg of proteins) were subjected to SDS–PAGE (Any kD™ Mini‐PROTEAN® Precast Protein Gels, BIORAD, Hercules, CA, USA) and transferred onto a 0.22 μ nitrocellulose membrane (Amersham™ Protran™, Global Life Sciences Solutions Operations, Buckinghamshire, UK) using Trans Blot® Turbo™ (BIORAD) and probed with the primary monoclonal antibodies: anti‐TrxR1; GR; Grx, Trx1, Gpx1/2, NAD(P)H quinone dehydrogenase 1 (NQO1), and superoxide dismutase 1 (SOD1) (Santa Cruz Biotechnology Inc., Santa Cruz, CA, USA). The WB detection was obtained using an ECL system with UVITEC (Alliance Q9 Advanced) equipment, and the densitometric analysis of WB bands was performed with NineAlliance software (Mini 9 17.01 version, Uvitec Alliance, Cambridge, UK), using glyceraldehyde‐3‐phosphate dehydrogenase (GAPDH) (Santa Cruz Biotechnology) as a loading control.

### 2.6 Nuclear fraction isolation and determination of Nrf2 levels

The method used for isolation of the nuclear fraction has been previously described.^36^ Caco‐2 cells seeded in T25 flasks were treated with 100 μM thiamine derivatives for 24 h. To obtain the nuclear fraction, cells were lysed at 4°C for 15 min with 100 μL of a solution containing 10 mM Hepes/Tris pH 7.9, 0.1 mM ethylene glycol‐bis(2‐aminoethylether)‐N,N,N′,N′‐tetraacetic acid (EGTA), 0.1 mM EDTA, 0.1 mM PMSF, 10 mM KCl, 1 mM NaF, and a protease inhibitor cocktail (Complete Roche, Mannheim, DE). Then, IGEPAL (5% final concentration) is added to the samples, subsequently mixed for 15 s, and centrifuged at 1000g for 10 min at 4°C. The resulting pellet (nuclear fraction) was dissolved in 20 mM Hepes/Tris (pH 7.9), 1 mM EGTA, 1 mM EDTA, 0.4 M NaCl, 0.1 mM PMSF, 1 mM NaF, a protease inhibitor cocktail, and centrifuged at 20,000g for 10 min at 4°C. Supernatants containing nuclear proteins (30 μg) were loaded onto a 10% SDS–PAGE and subjected to WB analysis to determine the levels of Nrf2 with proliferating cell nuclear antigen (PCNA) (Santa Cruz Biotechnology) as loading control. Densitometric analysis was performed using the Nine Alliance software as reported above.

### 2.7 Estimation of total thiol groups

Total cellular thiol groups were measured with the Ellman's assay.^37^ Briefly, cells (1 × 10^5^ cells/well) were pre‐incubated with 1 μM auranofin (AF) or 30 μM β‐chloro‐nitrosourea (BCNU, carmustine) for 1 h before treatment with 100 μM thiamine disulfides for 5 h. Then, cells were washed twice with PBS and lysed with 1 mL of ice‐cold 7.2 M guanidine in 0.2 M Tris–HCl buffer (pH 8.1) and 1 mM EDTA. The titration of free thiols after addition of 30 mM DTNB was monitored spectrophotometrically at 412 nm. The obtained values were normalized for the protein content measured according to Bradford.^38^

### 2.8 Assessment of ROS production

ROS production was assessed by using the fluorogenic probe 5‐(and 6)‐chloromethyl‐dichlorohydrofluorescein diacetate (CM‐H_2_DCFDA) (Molecular Probes, Thermo Fisher Scientific, Waltham, MA, USA). Caco‐2 cells (5 × 10^3^) were seeded in a 96‐well plate and, after 48 h, were treated for 24 h with 100 μM thiamine disulfide derivatives. Then, cells were washed in PBS 1×/10 mM glucose and loaded with 10 μM CM‐H_2_DCFDA for 20 min in the dark at 37°C. Afterwards, cells were washed and, where indicated, 300 μM TbOOH was added. Fluorescence increase was estimated on a plate reader (Tecan Infinite® M200 PRO) at 485 nm (*λ* excitation) and 527 nm (*λ* emission) for 120 min.

### 2.9 Mitochondrial respiration and glycolysis stress test assay in Caco‐2 cells treated with the thiamine disulfide derivatives

Cellular respiration and glycolysis were determined with the Seahorse XFe24 Analyzer (Agilent Technologies, Santa Clara, CA, USA) following the Cell Mito Stress Test and Glycolysis Stress Test protocols. For mitochondrial respiration, Caco‐2 cells were seeded (2 × 10^5^ cells/well) and grown in complete medium. Afterwards, cells were treated with 100 μM thiamine disulfide derivatives for the indicated times. Before starting the experiment, the medium was replaced with XF DMEM assay medium (pH 7.4), supplemented with 10 mM glucose, 1 mM sodium pyruvate, and 2 mM glutamine, and the cells were subjected to oxygen consumption analysis at 37°C. Three measurements of the basal respiration were performed, followed by the sequential injections of 1 μM oligomycin, 0.5 μM carbonyl cyanide *p*‐trifluoromethoxyphenylhydrazone (FCCP), and the combination 1 μM antimycin A plus 1 μM rotenone. For glycolysis estimation, Caco‐2 cells were harvested in the same conditions and treated with 100 μM thiamine disulfide derivatives for 24 h. Then, the medium was changed to XF DMEM assay medium (pH 7.4) supplemented with 2 mM glutamine, and cells were incubated in a non‐CO_2_ incubator at 37°C for 1 h before performing the assay. The Seahorse XFe24 Analyzer was calibrated, and the assay was performed using glycolytic stress test assay protocol as suggested by the manufacturer. Sequential injections of 10 mM glucose, 1 μM oligomycin, and 50 mM 2‐deoxy‐glucose (2‐DG) were carried out, and three measurements were done after each injection. At the end of the experiment, for data normalization, cells were lysed with 50 μL RIPA buffer and subjected to protein estimation.^31^

### 2.10 Statistical analysis

All the experimental data reported are the mean, with their respective Standard Deviations (SD), of at least three experiments. Analysis of variance (ANOVA) was performed with Tukey–Kramer method utilizing INSTAT 3.3 (Graph‐Pad) software. A *p* value <0.05 was considered significant.

## 3 RESULTS

### 3.1 Glutathione and thioredoxin systems reduce the thiamine disulfide derivatives

**FIGURE 1:**
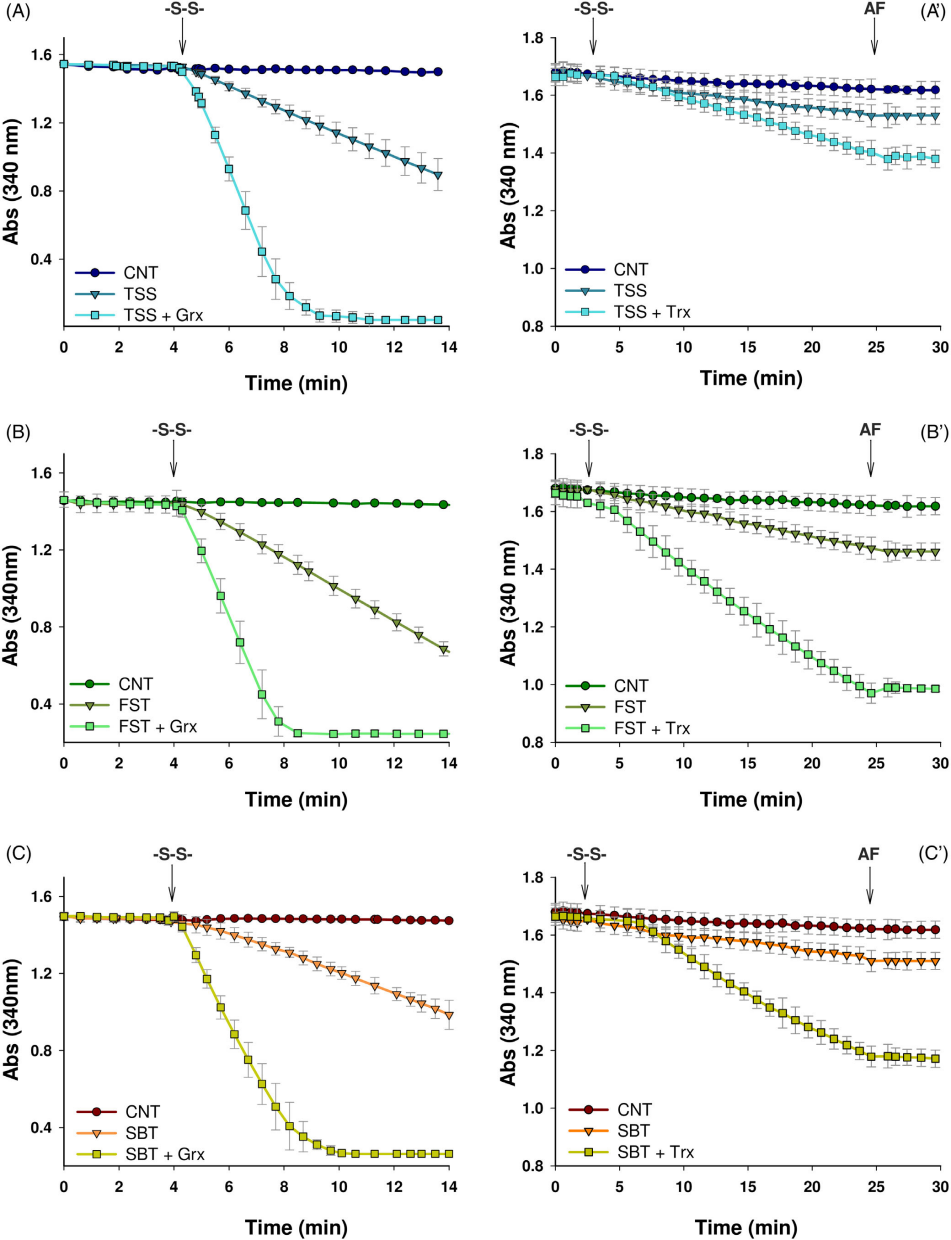
In vitro, enzymatic reduction of thiamine disulfide derivatives. On the left column (A–C) glutathione reductase activity was measured as indicated in Section 2 in the presence of 0.3 mM NADPH, 70 nM glutathione reductase, 0.1 mM GSH and, when present, 0.42 μM glutaredoxin (Grx). Where indicated (‐S‐S‐), 1 mM TSS (A), FST (B), and SBT (C) were added. On the right column (A′–C′), thioredoxin reductase activity was estimated in the presence of 0.3 mM NADPH, 72 nM thioredoxin reductase, and, when present, 7 μM thioredoxin (Trx). As specified (‐S‐S‐), 0.5 mM TSS (A'), FST (B′), SBT (C′) and, where indicated, 5 μM auranofin (AF) were added. Mean ± SD of at least three experiments was evaluated.

The glutathione system is constituted by the redox sequence NADPH, GR, and glutathione. NADPH, through GR, maintains glutathione in the reduced form that, in turn, delivers electrons to several enzymes, including Grx and Gpx. The Trx system is formed by NADPH, TrxR, and Trx, in a sequence where NADPH reduces TrxR which further transfers reducing equivalents to Trx. The latter acts as a reducing agent of several factors, such as peroxiredoxin, which decomposes H_2_O_2_ to water. Trx acts also as a general disulfide reductase.^39^ First, as apparent in Figure 1, thiamine disulfide, fursultiamine, and sulbutiamine can be reduced by both the Grx and Trx systems. GSH, largely abundant in any cell, is able to reduce the three disulfides examined as, once oxidized after interaction with the three disulfides derived from thiamine, is maintained constantly reduced by the presence of GR. As can be observed in the left column of Figure 1, reduction of 1 mM TSS (A), FST (B), and SBT (C) (collectively indicated as ‐S‐S‐) occurs only in the presence of glutathione, thus they are not direct substrates of GR. In addition, the reaction between the thiamine disulfide derivatives and GSH is markedly accelerated by the addition of Grx, which should act on the mixed disulfides formed between glutathione and thiamine. Then, we evaluated the action of the Trx system on the three disulfides (Figure 1A'–C′). The thiamine derivatives emerge as direct substrates of TrxR as NADPH oxidation was observed both in the presence and in the absence of Trx (Figure 1A'–C′), although at a lower rate. After about 25 min, the addition of AF, a specific and efficient inhibitor of the selenoenzyme TrxR,^40^ determined a complete stop of NADPH consumption, indicating that the reductive reaction was due solely to TrxR activity. Comparing the three disulfides, we also observed that all them can be reduced, but the activity rate of TrxR is particularly effective on FST (Figure 1B′). Therefore, both the glutathione/Grx and the Trx systems are able to reduce the three thiamine disulfide derivatives.

### 3.2 Impact of the thiamine disulfide derivatives on cell redox homeostasis

Considering the pharmacological relevance of the thiamine derivatives, it seemed of interest to explore their effects on the cellular redox state. Thus, we next moved our research to investigate the cellular oxidant/antioxidant balance in the presence of the three thiamine derivatives using the Caco‐2 cell line, which mimics the intestinal epithelium.

#### 3.2.1 Caco‐2 cell viability in the presence of thiamine disulfide derivatives

**FIGURE 2:**
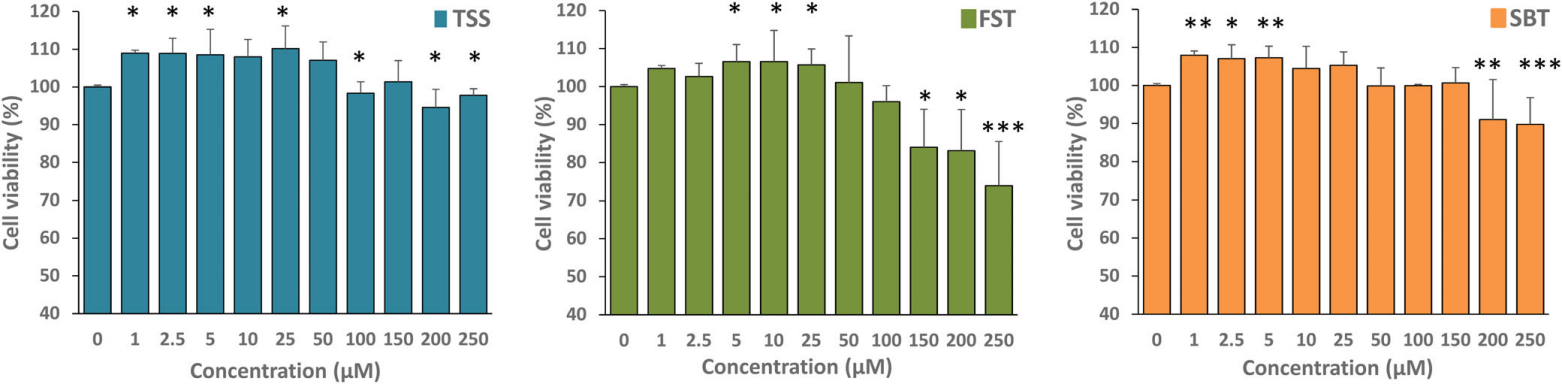
Cell viability in Caco‐2 cells in the presence of thiamine disulfide derivatives. Cell viability was determined with the 3‐[4,5‐dimethylthiazol‐2‐yl]‐ 2,5‐diphenyltetrazolium bromide reduction assay. Caco‐2 cells (1 × 10^4^) were treated with increasing concentrations of the thiamine disulfide derivatives for 24 h. Values reported are the mean of three independent experiments. ***p < 0.001; **p < 0.01; *p < 0.05.

First, Caco‐2 cells were treated with increasing concentrations of the thiamine disulfide derivatives in order to check their effect on cell proliferation using the MTT test. As reported in Figure 2, all the compounds displayed an initial increase in viability, which, however declined at higher concentrations. In particular, FST at concentrations larger than 100 μM led to a marked diminution of viability, as apparent by the comparison with the untreated control. Then, we proceeded with the subsequent experimentation using the three compounds at 100 μM concentration.

#### 3.2.2 Effects of the thiamine derivatives on Caco‐2 cells total thiols and ROS production

**FIGURE 3:**
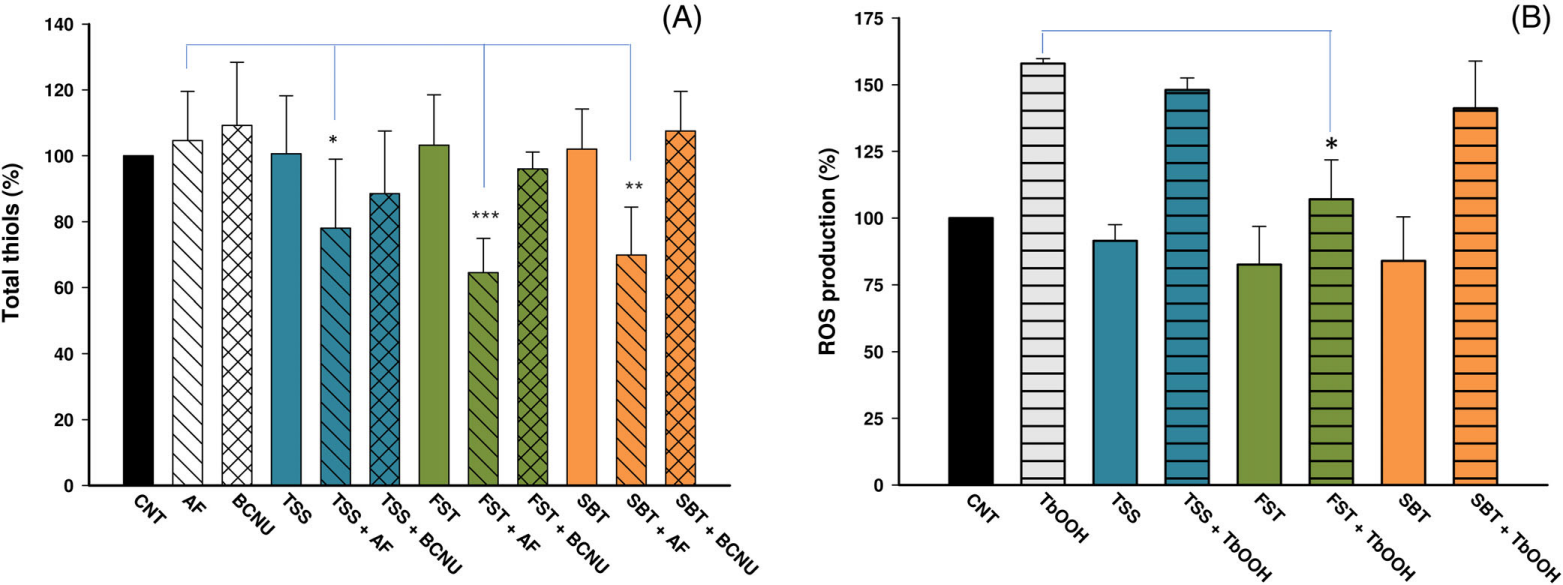
Effect of the thiamine disulfide derivatives on total thiols and reactive oxygen species (ROS) production. (A) Where indicated, Caco‐2 cells were treated for 1 h with 1 μM auranofin (AF) or 30 μM β‐chloro‐nitrosourea (BCNU). Then, 100 μM thiamine disulfide derivatives were added, for an overall time of 6 h. Total ‐SH groups were titrated with the 5,5′‐dithiobis(2‐nitrobenzoic acid) assay as described in Section 2 and expressed as a percentage of the control (CNT). (B) ROS production was estimated in Caco‐2 cells pretreated with 100 μM thiamine disulfide derivatives for 24 h. Then, cells were loaded with 10 μM CM‐H_2_DCFDA. To induce oxidative stress, cells were subjected to 300 μM *tert*‐butyl hydroperoxide (TbOOH) addition. Values reported are the mean of three independent experiments. Data are expressed as a percentage of the values observed in the control group. ***p < 0.001; **p < 0.01; *p < 0.05.

Caco‐2 cells were treated with TSS, FST, and SBT to estimate the redox state of the cell. Thiamine disulfide derivatives alone did not show any effect on total thiol groups, indicating that they were efficiently reduced by both glutathione and Trx systems. To confirm this hypothesis, we carried out the experiments in the presence of AF, a well‐known inhibitor, at submicromolar level, of TrxR, as previously stated, or in the presence of BCNU, a chemotherapeutic agent acting as an inhibitor of GR.^41^ Cells were pretreated with 1 μM AF or 30 μM BCNU, and after 1 h, the thiamine derivatives were added for a 5 h treatment. As reported in Figure 3A, at the indicated concentrations, AF and BCNU alone did not show any significant alteration of total thiol groups that, on the contrary, markedly decreased after treatment with thiamine disulfides in combination with AF. However, BCNU in combination with TSS, FST, and SBT did not exhibit such a large effect. This result seems to indicate that in the cell context, TrxR plays a major role in the reduction of thiamine‐derived disulfides, as these compounds, if not reduced to their thiol form by TrxR, can potentially alter the cellular redox balance. Therefore, with the combined contribution of the Trx and glutathione systems, thiamine disulfide derivatives are rapidly reduced in the cellular environment and not only they do not determine an imbalance on total thiols, but they are also capable of preventing induced oxidative stress, as apparent in Figure 3B. Cells were treated with 100 μM thiamine disulfide derivatives for 24 h, then oxidative stress was stimulated by the addition of 300 μM TbOOH, and the amount of ROS was detected by the fluorescent probe CM‐H_2_DCFDA. As depicted in Figure 3B, thiamine derivatives slightly reduced the basal level of ROS production, but in the presence of TbOOH, they were able to partially prevent oxidative stress. This effect was significantly evident in the presence of FST, as shown by the markedly reduced fluorescence values. Further, ROS production under conditions similar to those used for the thiol group assay where AF was present was estimated (Figure S1A). To this purpose, Caco‐2 cells (5 × 10^3^) were first incubated for 1 h with 1 μM AF, and subsequently 100 μM FST was added for a total of 6 h. Then, cells were washed and incubated with 10 μM CM‐H_2_DCFDA as reported in Figure S1A. Similarly to what was observed for total thiols (Figure 3A), the preincubation with AF, determined a significant increase in ROS levels as FST is no longer reduced by the TrxR thiol redox system and consequently does not exert its protective effect.

#### 3.2.3 Thiol redox enzyme activities after cell treatment with thiamine disulfide derivatives

**FIGURE 4:**
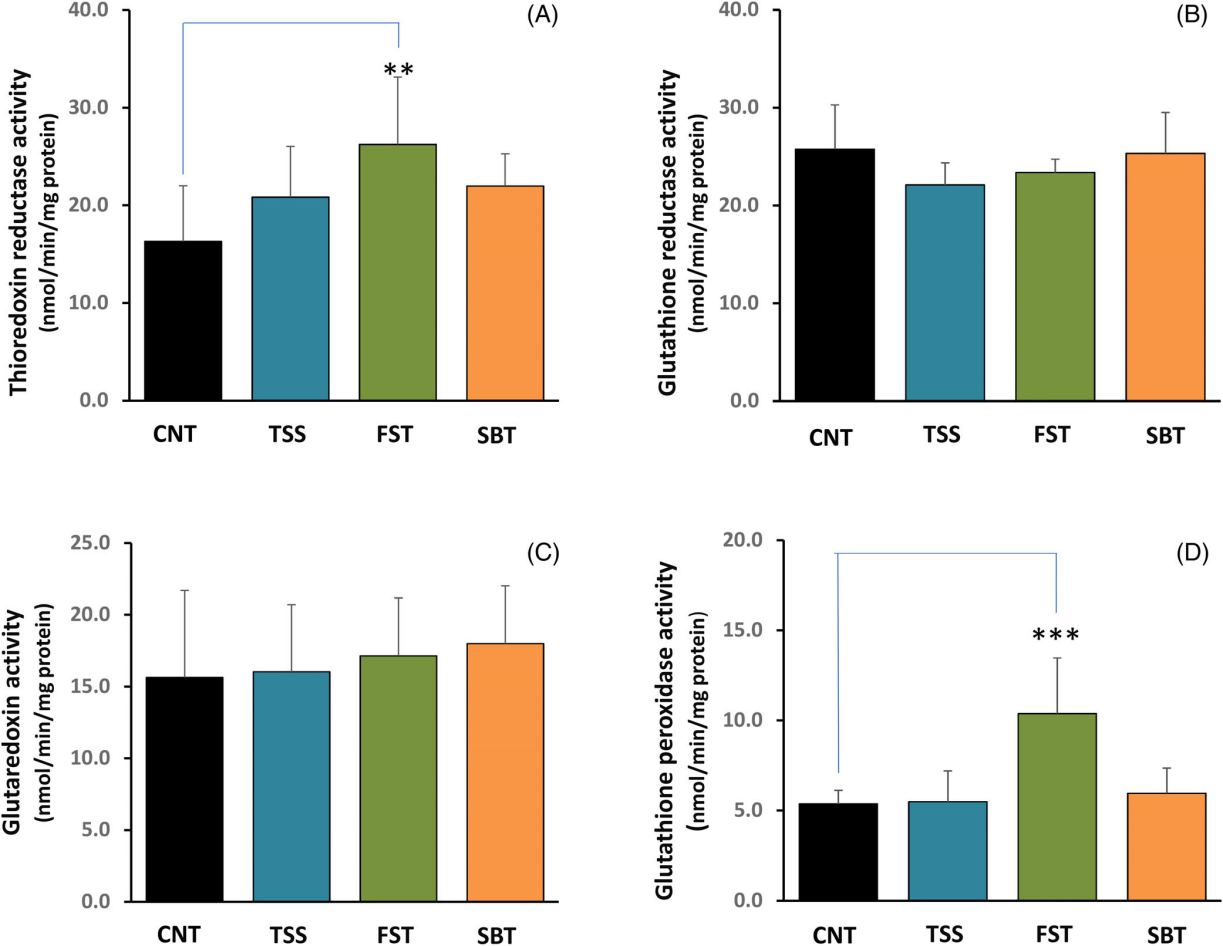
Enzyme activities in lysates of Caco‐2 cells treated with thiamine disulfide derivatives. (A) thioredoxin reductase, (B) glutathione reductase, (C) glutaredoxin, and (D) glutathione peroxidase. Cells were treated with 100 μM thiamine disulfides for 24 h and processed as described in Section 2. Total thioredoxin reductase activity was measured at 412 nm with the 5,5′‐dithiobis(2‐nitrobenzoic acid) assay, while glutathione reductase, glutaredoxin, and glutathione peroxidase were followed at 340 nm by a decrease of NADPH absorbance as described in Section 2. Values reported are the mean ± SD of three independent experiments, ***p < 0.001; **p < 0.01.

The effects of thiamine disulfide derivatives on the activities of thiol redox enzymes potentially involved in their reduction were studied by treating Caco‐2 cells (0.5 × 10^6^) with 100 μM thiamine disulfides, followed by specific activity estimation of TrxR, GR, Grx, and Gpx in the lysates. As reported in Figure 4, TrxR activity was increased in the presence of the three thiamine derivatives (Figure 4A). FST was particularly effective to determine a large increase of TrxR activity and was also able to rise Gpx activity, while TSS and SBT were ineffective (Figure 4D). However, GR (Figure 4B) and Grx (Figure 4C) showed no significant differences with respect to the untreated controls (CNT) with the three disulfides.

#### 3.2.4 Nrf2 nuclear translocation and expression of antioxidant enzymes in the presence of thiamine disulfide derivatives

**FIGURE 5:**
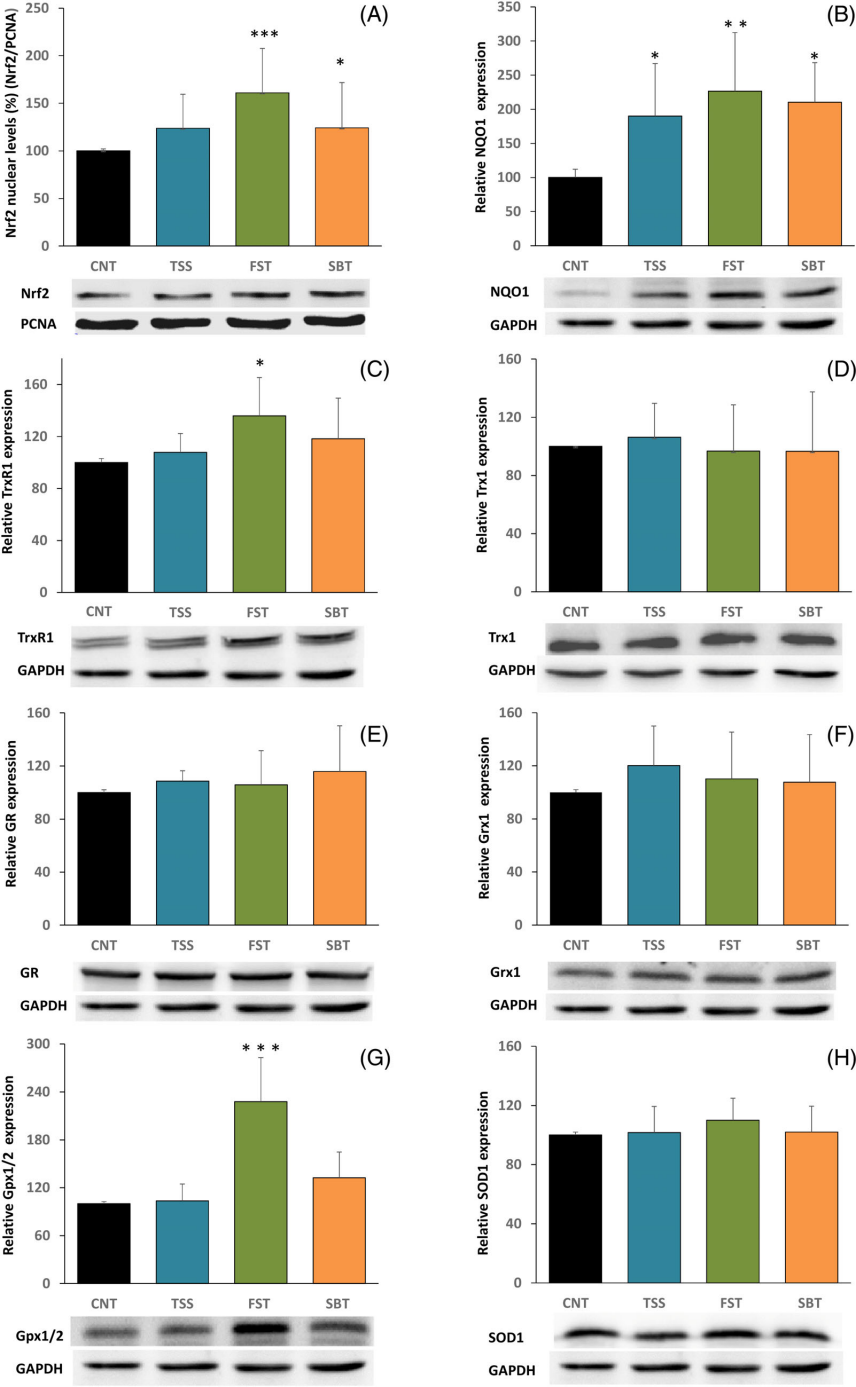
Activation of the Kelch‐like ECH‐Associating protein 1‐nuclear factor erythroid 2 related factor 2 (Nrf2)‐antioxidant response element pathway and expression of related enzymes in cells treated with the thiamine disulfide derivatives. (A) To determine Nrf2 translocation to the nucleus, the nuclear fraction obtained from Caco‐2 cells treated with 100 μM TSS, FST, and SBT was subjected to Western blot. The quantitative analysis was normalized using proliferating cell nuclear antigen (PCNA) as nuclear loading control. (B–H) Expression of NAD(P)H quinone dehydrogenase 1 (NQO1), TrxR1, Trx1, glutathione reductase (GR), Grx1, Gpx1/2 and superoxide dismutase 1 (SOD1), respectively. Aliquots of cell lysates treated with 100 μM TSS, FST, and SBT and subjected to WB were estimated by densitometric analysis performed using glyceraldehyde‐3‐phosphate dehydrogenase (GAPDH) as loading control. Values are indicated as mean ± SD of three independent experiments. ***p < 0.001; **p < 0.01; *p < 0.05. Gpx, glutathione peroxidase; Grx, glutaredoxin; TrxR, cytosolic thioredoxin reductase.

As long as GPx and TrxR are enzymes whose transcription is regulated by the Keap1/Nrf2 system,^42^ we investigated this signaling pathway after cell incubation with the thiamine disulfide derivatives. The translocation of Nrf2 from the cytosol to the nucleus was first measured and its levels were determined by WB analysis in the nuclear fraction (50 μg) to assess the activation of the Keap1‐Nrf2 pathway. As reported in Figure 5A, we observed an increase in nuclear translocation, especially evident in the presence of FST. The bars indicate the ratio of Nrf2 to PCNA, used as a loading control (mean of three different experiments). Subsequently, the expression of several enzymes directly involved in Nrf2 activation was measured. Cells were treated under the same conditions described above, and aliquots of the obtained lysates (30 μg) were subjected to WB analysis. First, we analyzed NQO1 protein levels, being one of the most responsive enzymes following the activation of Nrf2. As reported in Figure 5B, its expression increased significantly after treatment of the cells with 100 μM thiamine disulfide compounds and the most active derivative was FST. Then, the expression of the enzymes involved in the thiol redox balance was tested. In particular, TrxR (Figure 5C) and Gpx1/2 (Figure 5G) showed an increase in protein abundance, especially in the presence of FST, in accordance with what was found with the estimation of nuclear Nrf2 translocation (Figure 5A). A little to no expression was observed for Trx1 (Figure 5D), GR (Figure 5E), Grx1 (Figure 5F), and SOD1 (Figure 5H), at least in our conditions.

#### 3.2.5 Effect of thiamine disulfide derivatives on mitochondrial respiration

**FIGURE 6:**
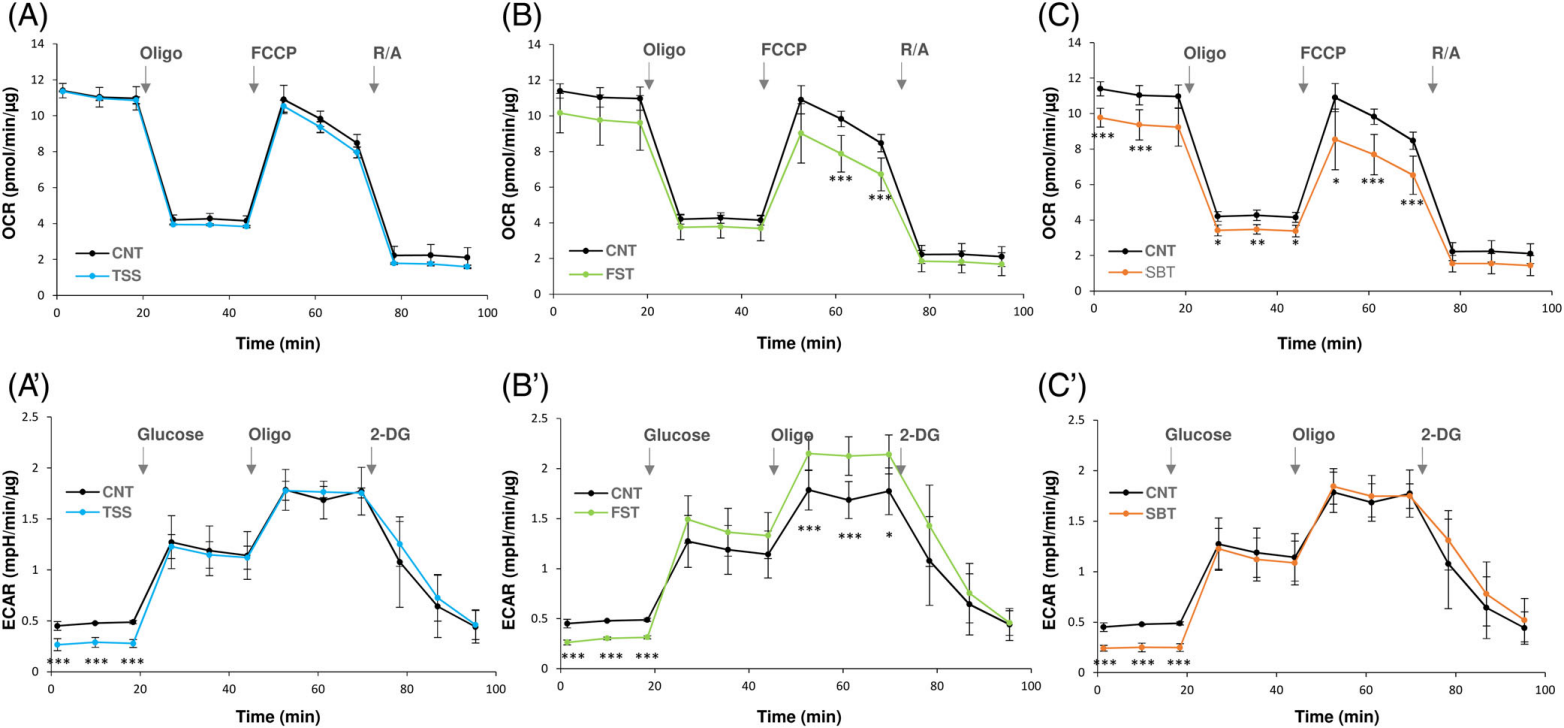
Oxygen consumption and glycolytic rates in cells treated with the thiamine disulfide derivatives. Caco‐2 cells were treated with 100 μM TSS (A, A′), FST (B, B′), or SBT (C, C′) for 24 h. Afterwards, the analysis of oxygen consumption rates (OCR) or extracellular acidification rates (ECAR) was performed using the Seahorse XFe24 analyzer as described in Section 2. (A–C) Basal respiration and respiratory capacity were evaluated after addition of 1 μM oligomycin (Oligo), 0.5 μM carbonyl cyanide p‐trifluoromethoxyphenylhydrazone (FCCP), and 1 μM antimycin A + 1 μM rotenone (R/A). (A′–C′) ECAR (glycolysis rate) was assessed after addition of 10 mM glucose, 1 μM oligomycin, and 50 mM 2‐deoxy‐glucose (2‐DG). The data are normalized for the protein content. Mean ± SD of three experiments is shown. ***p < 0.001; **p < 0.01; *p < 0.05.

Finally, to better understand the effects of thiamine disulfide derivatives on specific biochemical pathways, we tested their impact on cell respiration. To this purpose, the mitochondrial oxygen consumption rates (OCR) and extracellular acidification rates (ECAR) were estimated with the Seahorse technique as described in Section 2. When Caco‐2 cells were treated with the thiamine derivatives for 2 h, no substantial changes of OCR were detected (data not shown), but some effects take place after 24 h treatment. As apparent in Figure 6A,A′, while TSS did not affect both OCR and ECAR, the treatment with FST determined a decrease of the maximal OCR and a concomitant increase of ECAR, indicating a partial reduction of the electron flux through the mitochondrial electron transport chain favoring a stimulation of the glycolytic pathway (Figure 6B,B′). Finally, cells incubated in the presence of SBT showed a decrease of OCR, but no effect on ECAR. Of note, when the OCR estimation was performed in the presence of FST with the concomitant inhibition of the Trx system by AF, an even stronger effect on the mitochondrial oxygen consumption could be observed with a drop of oxygen consumption, especially in the uncoupled respiration (Figure S1B).

## 4 DISCUSSION

As reported in Section 1, thiamine can undergo opening of the thiazole ring generating a thiol species, followed by disulfide formation.^12^, ^17^ Therefore, taking advantage of these properties, several thiamine derivatives in the thioester or disulfide form and more bioavailable than thiamine were prepared.^12^ Cellular disulfides are in equilibrium with thiol groups by a thiol‐disulfide exchange and, in addition, specific enzymes such as those of the glutathione and Trx systems are able to reduce the disulfides back to thiols, exerting control over the thiol redox state.^39^, ^43^, ^44^ In plants such as *Arabidopsis thaliana* subjected to abiotic stress, an accumulation of thiamine and thiamine pyrophosphate due to enhanced expression of thiamine biosynthetic enzymes was observed. The same plant subjected to paraquat‐induced stress showed protection against oxidant challenge when exogenous thiamine was supplemented.^45^ Notably, under these conditions, the authors did not find accumulation of thiochrome (an irreversible end product of thiamine) and what is more, after supplementation with thiamine, the content of the latter did not decrease significantly when compared to the control without paraquat, suggesting a potential recycling of the oxidized disulfide form of thiamine.^45^ These results underline the efficiency of the disulfide‐reducing systems in maintaining the thiol redox equilibrium in which thiamine derivatives, especially during oxidative stress, are involved. The reduction of thiamine disulfide derivatives has been mainly attributed to GSH,^21^, ^22^ the most abundant low molecular weight cell thiol. Accordingly, the three thiamine disulfide derivatives examined in this work (TSS, FST, and SBT) are stoichiometrically reduced in the presence of glutathione maintained in its reduced form by GR and NADPH (data not shown). Moreover, on the basis of previous findings,^46^, ^47^, ^48^ we observed that this reaction is markedly stimulated by Grx a well‐known factor constituent of the glutathione/Grx system and involved in the thiolation/dethiolation processes in connection with NADPH, GSH, and GR.^43^ Kohno et al.^48^ found that benzoyl thiamine propyl disulfide was slowly reduced by GSH, but the reaction was markedly increased by the addition of a liver extract. They also purified an enzyme, particularly active in the liver, considered to specifically act on the disulfide‐type thiamine derivatives. Later, Eriksson and Guthenberg^46^ found that the enzyme thioltransferase acted as a catalyst in the GSH‐thiamine disulfide exchange reaction and was able to reduce benzoyl thiamine propyl disulfide. It has to be taken into account that currently thioltransferase is called Grx and that, on the basis of enzyme properties, amino acid sequence, and immunochemical features, the two terms (thioltransferase and Grx) identify the same protein.^49^ However, according to our results, the glutathione/Grx system does not appear to play an exclusive role in the reduction of the disulfides derived from thiamine. We also examined the Trx system, previously shown to act on several low molecular weight and protein disulfides.^47^, ^50^ We found that the complete Trx system (NADPH, Trx, and TrxR) is able to reduce the thiamine disulfides but, in the absence of Trx, also the simple combination of TrxR + NADPH can catalyze the reduction of thiamine disulfides (Figure 1). Therefore, at variance with GR, which turns out to be highly specific for GSSG and hence ineffective on the thiamine disulfides, TrxR is able to directly act on the reduction of the disulfide moiety of the examined compounds.

The ability of the thiol‐reducing systems to act on thiamine‐derived disulfides was also examined in cultured Caco‐2 cells. The addition of the three disulfide derivatives does not alter the amount of the total thiols estimated with the Ellman's assay. This lack of change in total thiol content may depend on the rapid formation of the thiamine cyclic form, as the open thiol form of thiamine, once formed, is very short lived^22^ and thus the thiols formed after reduction of the disulfides by the Trx and Grx systems do not substantially contribute to the low molecular weight thiol pool, at least at the concentration used. Therefore, the total thiol redox balance does not change substantially.

Previously, it has been shown^41^ that, in isolated mitochondria, AF (a strong inhibitor of TrxR) and BCNU (an inhibitor of GR) either stimulate H_2_O_2_ production or suppress its removal both due to the inhibition of TrxR or GR, respectively. In Caco‐2 cells treated with the three thiamine‐derived disulfides in the presence of AF, the total estimated thiols markedly decreased, largely below the basal level observed in the control (Figure 3A). A less powerful effect is also exhibited by BCNU, indicating also the involvement of the glutathione/Grx system in the reduction of the thiamine‐derived disulfides. Of note, both AF and BCNU at the concentrations employed and in the absence of the disulfides, are ineffective in modifying total thiols. Therefore, the inhibition, particularly of the Trx system, and the consequent lack of disulfide reduction prompt the oxidation capacity of the added disulfides on both protein and low molecular weight thiols, as observed from the decrease of the measured total thiol groups. When oxidation was forced in Caco‐2 cells by adding TbOOH, which acts as a substrate of Gpx and peroxiredoxin besides eliciting the formation of free radical species, the thiamine disulfide derivatives showed protection against oxidative stress. Previously,^51^ mouse neuroblastoma cells (Neuro2a) grown in thiamine‐restricted medium and treated with paraquat, a redox cycler producing superoxide and hence hydrogen peroxide, were shown to obtain protection from oxidative stress when sulbutiamine, benfotiamine (BFT), or large concentrations of thiamine were present. This shows an antioxidant action exerted by these molecules and not mediated by stimulation of thiamine diphosphate‐dependent enzymes. Furthermore, in *A. thaliana* seedlings, the prooxidant action of paraquat was prevented by thiamine supplementation,^45^ as already reported.

The effect of the three disulfides on the viability of Caco‐2 cells was tested, first of all to set the proper concentrations to be employed in the further experimentation. However, in Figure 2, it is clearly apparent that the three thiamine‐derived disulfides increased cell viability at low concentrations but decreased it at higher concentrations, indicating a biphasic effect that in turn suggests their potential effectiveness as signaling factors. Several reports indicate that the derivatives of thiamine can act as signaling molecules, especially by activating the Keap1‐Nrf2‐ARE pathway. Majid et al.^52^ found that in the transformed retinal ganglion cell line (RGC‐5), sulbutiamine stimulates catalase activity and increases heme oxygenase (HO‐1) and Nrf2 levels. More recently, BFT was shown to activate the Nrf2‐ARE pathway in a transgenic mouse model of tauopathy.^53^ In human neuroblastoma SH‐SY5Y cells stably expressing Neh2‐luc reporter, BFT and particularly its metabolites were shown to exert a potent activation in a process where thiamine is inactive.^53^ The effect was attributed to a Nrf2‐displacement mechanism essentially confirmed by a high docking score obtained by molecular modeling.^53^ The activation of Nrf2 transcription factor can be induced considering the large presence of reactive SH groups in the Keap‐1 protein that can undergo a thiol‐disulfide exchange with the formation of disulfides or mixed disulfides enhancing the activation of the transcription factor Nrf2.^42^ For instance, diallyldisulfide^54^ and oxidized lipoic acid^55^ were shown to be good activators of Nrf2. Therefore, it is tempting to speculate that the disulfide derivatives of thiamine may act on Keap1 by a thiol‐disulfide exchange and hence activate the transcription factor Nrf2. According to our results, the three disulfide derivatives are able to increase the nuclear level of Nrf2 and stimulate the expression of NQO1, TrxR, and, by fursultiamine, also of Gpx1, while the other enzymes of the thiol redox system are scarcely stimulated. Notably, the stimulated enzymes TrxR and Gpx1 are the two selenium‐dependent enzymes of these systems. Also, their estimated enzymatic activities were increased. In the case of Gpx, we found a stimulation solely with FST treatment, and this may depend on the asymmetrical nature of this disulfide. According to our results and in particular, considering both the byphasic effect observed in the viability tests (Figure 2) and the activation of the Nrf2‐dependent transcription system (Figure 5), the thiamine‐derived disulfides can be included among the factors able to stimulate the network of the vitagenes, which are a number of protective genes particularly sensitive to redox regulation and able to preserve cell homeostasis.^56^, ^57^

Regarding the impact of the thiamine derivatives on cell metabolism, we observed different effects of the three compounds on the mitochondrial respiratory capacity and on the glycolytic rates (Figure 6). SBT and FST show a decrease in mitochondrial oxygen consumption, while FST was the only one able to increase glycolysis.

In conclusion, our results describe the reduction pathways of three major derivatives of thiamine: thiamine disulfide, sulbutiamine, and fursultiamine. These disulfides, in addition to their capability of maintaining a high level of thiamine in tissues, may also act in the cell signaling pathways as shown by increased viability at low concentrations, Nrf2 activation, and modulation of the thiol redox balance. However, higher concentrations in the absence of an efficient reducing system might shift the thiol redox balance to a more oxidized condition and alter cell functions as observed after addition of AF. In fact, while respiration was modestly affected by these disulfides, inhibition of TrxR by AF in the presence of FST almost completely prevented the uncoupled respiration.

As the compounds studied are already utilized as drugs or food supplements, the reported results can be considered for a potential translation to clinical applications. Although further research is needed, an efficient nutritional or pharmacological supply of thiamine derivatives can be taken into account for interventions in unbalanced redox conditions and in the control of chronic inflammation.

## AUTHOR CONTRIBUTIONS

Conceptualization: AB and MPR, Methodology: AF, VS, and FT. Formal analysis: AF, FT, and VS. Investigation: AF, VS, and FT. Data curation: AF, VS, FT, and MPR. Writing—original draft preparation: AB and MPR, Writing—review and editing: AB and MPR. Funding acquisition: MPR. All authors have read and agreed to the published version of the manuscript.

## CONFLICT OF INTEREST STATEMENT

All the authors confirmed no conflict of interest.

## Supporting information


**Data S1.** Supporting Information.
