# Supplementary material for: Thiamine disulfide derivatives in thiol redox regulation: Role of thioredoxin and glutathione systems
Source: Biofactors. 2024 Sep 20;51(1):e2121. doi: 10.1002/biof.2121 (PMC11681303; doi:10.1002/biof.2121)
Supplement: Supplementary file 1 — Data S1. Supporting Information. [file BIOF-51-0-s001.docx]

**Supplementary Information**

**THIAMINE DISULFIDE DERIVATIVES IN THIOL REDOX REGULATION: ROLE OF THIOREDOXIN AND GLUTATHIONE SYSTEMS**

**Alessandra Folda^1^, Valeria Scalcon^1^, Federica Tonolo^2^, Maria Pia Rigobello^1^* and Alberto Bindoli ^1,3^***

1 Department of Biomedical Sciences, Via Ugo Bassi 58/B, 35131 Padova, Italy

2 Department of Comparative Biomedicine and Food Science, Viale dell’Università 16, 35020 Legnaro (PD), Italy

3Institute of Neuroscience (CNR), viale G. Colombo 3, 35131, Padova, Italy

Running title: **Thiamine disulfide derivatives in thiol redox regulation**

*Corresponding author: Maria Pia Rigobello

*Co-corresponding author: Alberto Bindoli


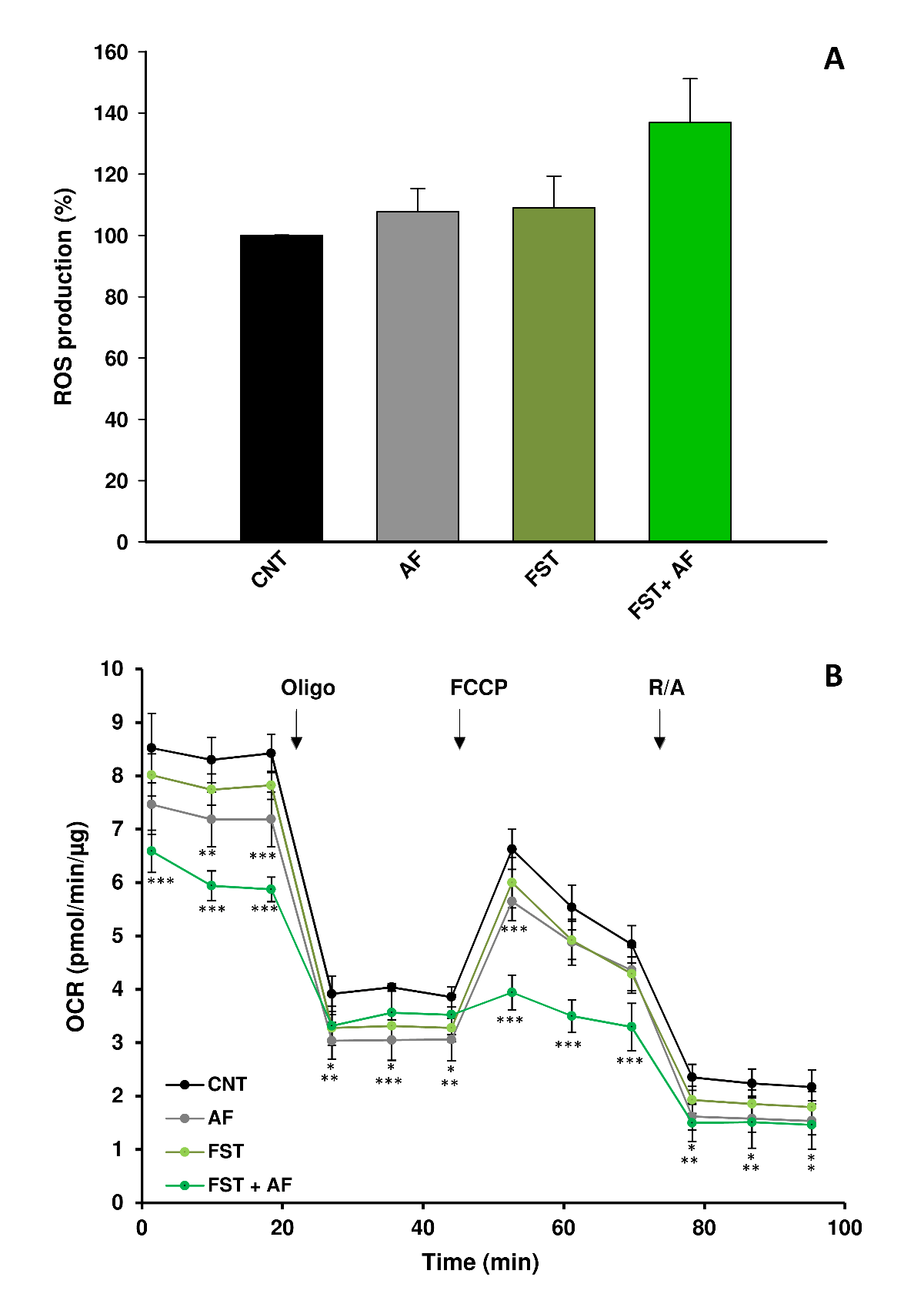


**Figure 1 SI**: **Estimation of endogenous ROS production and OCR in Caco-2 cells in the presence of FST and with the combination FST/AF.** A: ROS production. Where indicated, cells (5 x 10^3^) were pre-treated with 1 μM AF (1 h) and then for 5 h with 100 μM FST. Fluorescence increase was estimated as indicated under Experimental procedures. B: Oxygen consumption rates (OCR). Caco-2 cells (2 x 10^4^) were treated, where indicated, with 1 μM AF and 100 µM FST in the same conditions of ROS production. The analysis of oxygen consumption rates was performed using the Seahorse XFe24 analyzer as described in the Experimental procedures. Basal respiration and respiratory capacity were determined after sequential addition of 1 µM oligomycin (Oligo), 0.5 µM FCCP and the combination of 1 μM antimycin A + 1 µM rotenone (R/A). Values reported are the mean of three independent experiments. *** p<0.001; ** p<0.01; * p<0.05.
